# Spatiotemporal dynamics of urban climate during the wet-dry season transition in a tropical African city

**DOI:** 10.1007/s00484-020-02061-1

**Published:** 2021-01-06

**Authors:** Peter Kabano, Angela Harris, Sarah Lindley

**Affiliations:** 1 Department of Geography, School of Environment, Education & Development, The University of Manchester, Manchester, UK; 2 Department of Urban and Regional Planning and Geo-information Management, Faculty of Geo-Information Science and Earth Observation (ITC), University of Twente, Enschede, The Netherlands

**Keywords:** Tropics, Urban climate, Seasons, Temperature, Humidity, Heat index, Surface moisture, Regression models

## Abstract

The Urban Heat Island effect has been the focus of several studies concerned with the effects of urbanisation on human and ecosystem health. Humidity, however, remains much less studied, although it is useful for characterising human thermal comfort, the Urban Dryness Island effect and vegetation development. Furthermore, variability in microscale climate due to differences in land cover is increasingly crucial for understanding urbanisation effects on the health and wellbeing of living organisms. We used regression analysis to investigate the spatial and temporal dynamics of temperature, humidity and heat index in the tropical African city of Kampala, Uganda. We gathered data during the wet to dry season transition from 22 locations that represent the wide range of urban morphological differences in Kampala. Our analysis showed that the advancement of the dry season increased variability of climate in Kampala and that the most built-up locations experienced the most profound seasonal changes in climate. This work stresses the need to account for water availability and humidity to improve our understanding of human and ecosystem health in cities.

**Supplementary Information:**

The online version contains supplementary material available at 10.1007/s00484-020-02061-1.

## Introduction

The replacement of natural vegetation and pervious land cover with impervious cover, and intensified human activity (e.g. heavier traffic) through urbanisation alter the cycling of materials and energy in the atmosphere and near-surface (Pataki et al. 2011; Pickett et al. 2011; Wu 2014). Artificial surfaces (e.g. concrete, asphalt and metal) and the compact nature of cities (i.e. high building density, tall buildings, narrow streets) promote the transformation of short wave radiation into heat and its retention (Landsberg 1981; Oke 1987), and restricts latent heat flux through evapotranspiration (Taha 1997; Weng et al. 2004; Feyisa et al. 2014; Duarte et al. 2015). Consequently, cities experience warmer temperatures in what is commonly referred to as the Urban Heat Island effect (Landsberg 1981; Voogt and Oke 2003; Heisler and Brazel 2010). The Urban Heat Island (UHI) effect has received considerable critical attention because of its adverse effects on public health and wellbeing through increased heat exposure (Heaviside et al. 2017). Traditionally, UHI studies involve comparing temperatures between urban and rural meteorological stations (Stewart and Oke 2012). However, heterogeneity in surface cover and structure within cities necessitates consideration of microscale climatic processes (Oke 2008; Roth 2012). Much research has been done to date to understand how individual aspects of urban environments (e.g. vegetation) influence urban climate (e.g. Feyisa et al. 2014; Duarte et al. 2015; Giridharan and Emmanuel 2018; Acero and Gonzalez-Asensio 2018). Additionally, the importance of examining the effect of land cover composition as a whole on urban climate has recently gained much attention (Stewart and Oke 2012), and regression modelling is increasingly used for this purpose (Johnson et al. 2020).

Humidity is an important climatic variable that influences the availability of surface moisture, energy budget, human thermal comfort and ecological systems (Hao et al. 2018; Luo and Lau 2019). Cities generally experience lower humidity than their rural surrounding in what is commonly referred to as the Urban Dryness Island (UDI) effect (Adebayo 1991; Lokoshchenko 2017; Yang et al. 2017). Limited water uptake due to impervious cover (Barnes et al. 2001; Whitford et al. 2001), higher potential evapotranspiration (PET) resulting from the UHI effect and low vegetation cover are the key factors that lead to the formation of the UDI (Hao et al. 2018; Luo and Lau 2019). The UDI also influences the UHI through evapotranspiration, indicating that the two variables are coupled (Lokoshchenko 2017; Hao et al. 2018). In the tropics where surface moisture and humidity are essential indicators of vegetation development (Archibald and Scholes 2007; Jochner et al. 2013; de Camargo et al. 2018), higher plant water requirements due to high PET in cities (Zipper et al. 2017) and UDI might restrict vegetation growth. However, a vast number of tropical urban climate studies have focussed on the UHI effect alone, and far fewer studies have looked at humidity (Giridharan and Emmanuel 2018). Moreover, several studies about the UDI effect have used the urban-rural dichotomy (e.g. Yang et al. 2017; Hao et al. 2018; Luo and Lau 2019), necessitating studying humidity at the neighbourhood (micro) scale.

In the tropics, UHI and UDI intensities weaken during the wet season (Adebayo 1991; Balogun and Balogun 2014; Ayanlade 2016; Ojeh et al. 2016). Roth (2007) suggested that increased thermal admittance of wetter soils in rural areas during the wet season could account for weak UHI intensities. An improved understanding of how moisture availability affects local climate and its variability in cities could be reached using meteorological observations at a high temporal resolution. In the tropics where leaf flush of trees in natural habitats occurs during the wet-dry season transition (de Camargo et al. 2018), information about variability of urban climate could be useful for understanding leaf flush dynamics in cities and how that impacts on thermal cooling and provision of shade for urban residents.

The past decade has seen a significant upward trend in the proportion of tropical UHI studies since the low estimate of 20% in 2007 (Roth 2007). However, there is a disparity in the number of tropical urban climate studies with respect to geographic regions. Far fewer studies have been done in Africa in comparison to Far East Asia, South Asia and South America (Giridharan and Emmanuel 2018). Moreover, far fewer studies have been done in moist tropical climate types (tropical rainforest, savanna wet and dry and tropical monsoon) in sub-Saharan Africa. Due to differences in water availability and temperature between tropical climate types (Kottek et al. 2006; Peel et al. 2007), a wide range of exemplar urban climate studies are needed from different tropical climate zones.

Africa is rapidly urbanising, and its urban population is expected to reach 1.26 billion by 2050 from estimates of 400 million (United Nations 2014). Urbanisation in Africa accounts for high losses of natural vegetation cover in cities each year (Yao et al. 2019), although vegetation is strongly depended upon for the provision of urban ecosystem functions like the mitigation of high all-year-round tropical temperatures (du Toit et al. 2018; Lindley et al. 2018). Understanding the climate of tropical African cities could provide information for designing climate-sensitive cities (Heisler and Brazel 2010; Pauleit et al. 2015).

In this paper, we used regression analysis to examine the influence of changes in surface moisture and land cover composition on temperature, humidity and heat index in Kampala, Uganda. Our main objectives were as follows:

- To determine the influence of changes in surface moisture on the local climate in Kampala. We anticipate that spatial variability in urban climate is dependent on changes in surface moisture and variability in local climate within the city increases with the advancement of the dry season
- To establish the relationship between land cover composition and urban climate

## Methods

### Study area

**Fig. 1:**
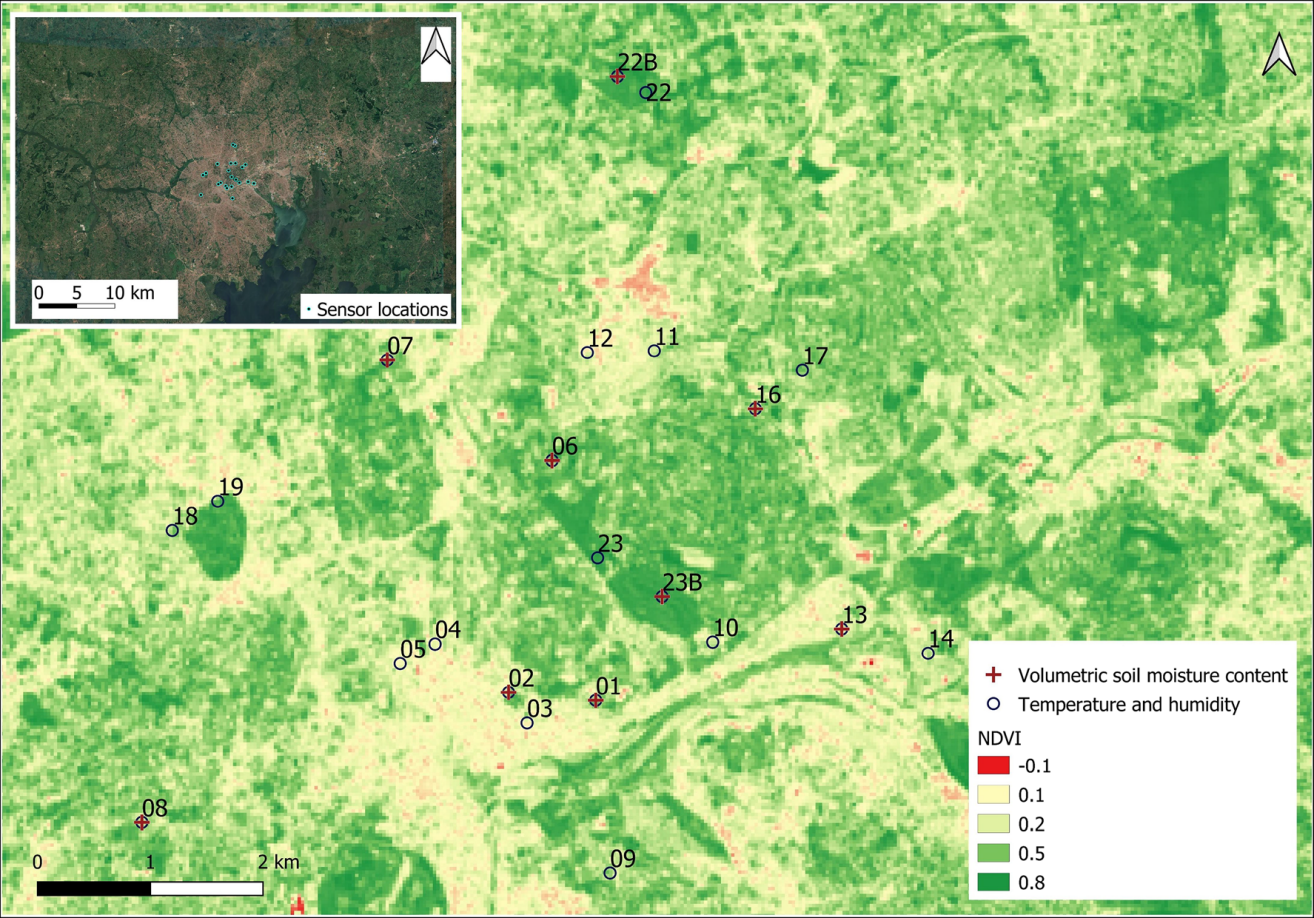
Location (numbered) of the urban climate monitoring sites and volumetric soil moisture content (crossed) in relation to the proportion of human-made features (and vegetation cover, as indicated by higher values of the NDVI (normalised difference vegetation index)). The image in the top left corner shows the selected sites in relation to Kampala’s urban extent

**Fig. 2:**
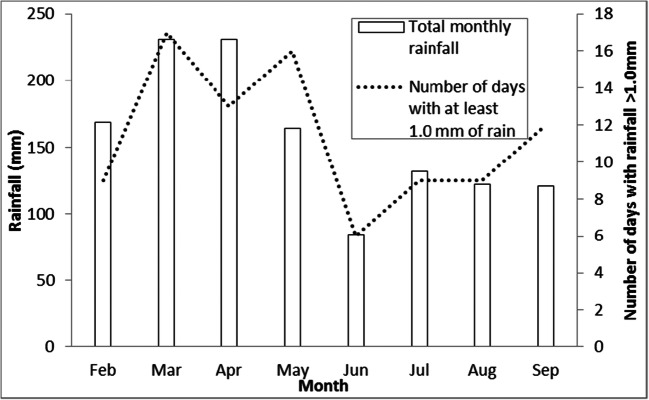
Kampala’s climate between February and September 2017, depicting monthly rainfall and number of days with more than 1 mm of rain

This study was undertaken in Kampala (located at 00° 18′ 49″ N, 32° 34′ 52″ E), the capital city of Uganda (Fig. 1), with a population density of about 8700 inhabitants/km^2^. Kampala has a tropical rainforest (equatorial) climate (Af Köppen climate classification) and experiences two rainy seasons per year, during March–May and September–November. March–May is the shorter of the two rainy seasons, but this season experiences the most torrential rains (approximately 169 mm). July receives the least rainfall (approximately 63 mm) throughout the entire year. In 2017, the dry season started in May and continued through September (Fig. 2). This study focused on 50 days spanning the wet-dry transition (i.e. Julian day of year (DOY): 100–150).

### Meteorological sites and data

We selected twenty-two sites with varying surface cover and structural characteristics (Fig. 1; Table 1) to account for variation in local climate (Stewart and Oke 2012) and variation in levels of exposure of biological organisms to the effects of urbanisation (Grimm et al. 2008; McCarthy et al. 2010; Pataki et al. 2011; Pickett et al. 2011; Wu 2014). Air temperature and relative humidity data were acquired at each site using i-button sensors (model DS1923, Maxim Integrated) housed in a radiation shield positioned at the height of 3 m above the ground. The loggers were individually factory calibrated in a NIST (National Institute of Standards and Technology)-traceable chamber before deployment in the field and assessed for drift during data collection. Each sensor collected temperature and relative humidity data at 30-min intervals.

**Table 1:** The categories of the degree of urbanisation and proportion of different land cover types (impervious cover, paved cover, buildings, trees and pervious cover) at each site. Locations used for observations of surface moisture are in italics

Degree of urbanisation	Location number (code)	Impervious cover (%)	Paved cover (%)	Buildings (%)	Trees (%)	Pervious (%)
High	05	84	49	35	5	11
	12	81	21	60	6	13
	04	80	35	44	9	11
	03	79	36	44	9	12
	11	69	37	32	11	21
	14	65	32	33	13	21
Medium	*13*	*59*	*39*	*21*	*11*	*30*
	10	59	39	21	21	19
	*01*	*51*	*32*	*20*	*37*	*12*
	09	49	17	32	24	26
	*02*	*48*	*39*	*9*	*30*	*22*
	17	44	22	22	28	28
	18	37	14	23	20	43
	*16*	*33*	*13*	*20*	*31*	*35*
	*08*	*33*	*16*	*17*	*18*	*49*
Low	*07*	*23*	*5*	*19*	*35*	*42*
	*06*	*19*	*10*	*9*	*15*	*65*
	19	19	11	8	31	50
	23	9	8	1	43	48
	22	5	4	2	50	45
	*22B*	*0*	*0*	*0*	*60*	*40*
	*23B*	*0*	*0*	*0*	*44*	*56*

As there is no perfect measure of humidity for comparison across sites (Adebayo 1991; Hao et al. 2018), we examined four humidity indicators, including relative humidity (RH), atmospheric water vapour pressure (Ea), specific humidity (*Q*), and vapour pressure deficit (VPD). The humidity measures were obtained as follows: First, we obtained saturated vapour pressure (hPa; hectopascals) as Es = 6.108 × exp[(17.27 × *T*)/(273.3.5 + *T*)], where *T* represents temperature (°C); vapour pressure (hPa) was acquired using the equation Ea = Es × RH/100; VPD in hPa was defined as the difference between Ea and Es: VPD = Es − Ea; specific humidity (*Q*, g/kg) was estimated using the equation *Q* = (622 × Ea)/(*P* − 0.378 × Ea), in which *P* is pressure in hPa.

A bi-quadratic function that estimates human heat exposure using temperature (*T*) and relative humidity (RH) observations was used to calculate heat index (HI), as follows:

$$ \mathrm{HI}={c}_1+{c}_2T+{c}_3\mathrm{RH}+{c}_4T\mathrm{RH}+{c}_5{T}^2+{c}_6{\mathrm{RH}}^2+{c}_7{T}^2\mathrm{RH}+{c}_8T{\mathrm{RH}}^2+{c}_9{T}^2{\mathrm{RH}}^2 $$

where *c*_1_ = − 42.379, *c*_2_ = 2.04901523, *c*_3_ = 10.14333127, *c*_4_ = − 0.22475541, *c*_5_ = − 0.00683783, *c*_6_ = − 0.05481717, *c*_7_ = 0.00122874, *c*_8_ = 0.00085282, and*c*_9_ = − 0.00000199 (Steadman 1979).

We obtained the daytime and nighttime (sunset 18:00 to sunrise 06:00) averages for each climatic variable each day at each location, and the daily mean and standard deviation across all sites. This way, the standard deviation was used to represent intra-urban climatic variability across time (Adebayo 1991). The time series for each meteorological variable are presented in the supplementary material (Fig A1 and Fig A2).

#### Characterisation of meteorological sites

The urban environment was characterised via an Object-Based Image Analysis classification of a WorldView3 satellite image (spatial resolution of 0.5 m) taken on 25/10/2016. Buildings, paved cover, pervious cover (grass and bare soils) and trees were classified and their proportion (percentage) at each site quantified within 200 m. Although a radius of 200 m has been recommended as the zone for attribution of local climate to land cover composition (Stewart and Oke 2012), the land cover in our candidate sites was heterogeneous. Therefore, the representativeness of an area of a 200-m radius for attributing local climate to land cover was compared to the effectiveness of a smaller area (radius of 100 m) with less land cover heterogeneity.

Each site was assigned to one of three categories representing the degree of urbanisation (i.e. high, medium and low) using hierarchical cluster analysis for the 100-m and 200-m land cover data separately. Analysis of similarity (ANOSIM) (Clarke 1993) was used to statistically examine whether urban climate varied significantly between the three categories of degree of urbanisation for the 100-m vs 200-m data. The ANOSIM indicated that the 100 m dataset showed more significant differences in urban climate between categories of the degree of urbanisation than the 200-m radius (*R* = 0.556 (*p* = 0.001) and *R* = 0.321 (*p* = 0.005); respectively). Therefore, subsequent analysis on the influence of landcover on urban climate was based on land cover within a radius of 100 m of each meteorological site (Table 1).

#### Data on surface moisture changes

**Fig. 3:**
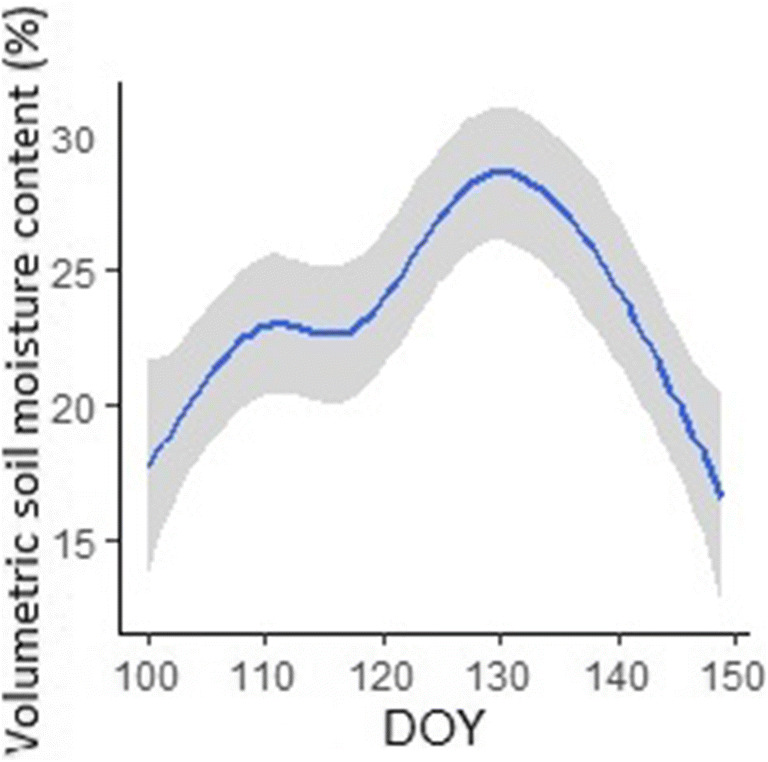
Temporal change in soil moisture across Kampala for delineation of the wet and dry seasons (DOY = 129). The error band shows the 95% confidence limits

Soil volumetric moisture content (VMC; Table 1) was measured in nine sites to relate changes in surface moisture to changes in urban climate and its variability across Kampala. Soil VMC (expressed as a percentage) was measured twice a week using a ThetaProbe (model ML3 ThetaProbe, Delta-T Devices), at five points at each site. The VMC data were temporally interpolated using a locally weighted regression (loess) model to derive a daily time series of surface moisture across Kampala (Fig. 3). The day of year (DOY) when VMC was greatest marked the end of the wet season and the start of the dry season (Fig. 3).

### Data analysis

#### Influence of soil moisture on urban climate

Linear regression modelling was used to determine the influence of changes in surface moisture on climate and its variability within Kampala. We used daily VMC aggregated across all sites as the predictor variable and the daily mean and standard deviation of urban climate (temperature, humidity and heat index) as response variables. Location 13 was dropped from the analysis of urban climate patterns due to gaps in data, but was used for VMC data. Linear mixed models (Baayen 2008) were used to determine the effect of degree of urbanisation (proportion of human-made features (paved cover and buildings)) on temporal changes in urban climate with the advancement of the dry season. The proportion of impervious cover (i.e. the proportion of buildings and paved cover) and Julian day (DOY) and their interaction were used as fixed effects (predictor variables). Each data point in each model represented the urban climatic data on a given day at a given location. Individual location was included as a random effect for correlated error terms caused by repeated measures taken at the same location. The significance of the full model was determined using a likelihood ratio test comparing the full model to a null model (lacking the temporal autocorrelation structure). Visual inspection of residuals plotted against fitted values revealed normally distributed and homogeneous residuals. The modelling was done in R using the “nlme” package (Pinheiro et al. 2018; R Core Team 2018) and the effects visualised with the R package “effects” (Fox and Weisberg 2018).

#### Influence of land cover composition

The relative influence of land cover composition on urban climate (nighttime temperature, humidity and heat index) averaged across the dry and wet seasons separately was assessed using an information-theoretic approach (Burnham et al. 2011). Regression models were formulated using combinations of indicators of land cover (i.e. the proportion of paved cover, pervious cover, buildings and trees) as predictors of urban climate for the wet and dry seasons separately. Collinearity between predictor variables was assessed by calculating variance inflation factors (“vif” function of the R package *car*) for each model, and variables with VIF > 3 subsequently removed from the models. The explanatory effect of other variables not covered under the scope of this study was accounted for by including a null model in each set of models. Model selection was undertaken using the MuMIn package in R (R version 3.5.0 (Barton 2018; R Core Team 2018)) to identify models with the simplest structure that best predicted urban climate. Ranking of models was performed using the Akaike Information Criterion (AICc) corrected for small samples. The significance of predictor variables was weak if the null model (intercept only) had a ΔAICc = 0. Models with ΔAIC < 2 were considered as potentially suitable models. Variables that best predicted urban climate were identified from the relative importance values (RIV) derived from the sum of Akaike weights (Burnham and Anderson 2003).

## Results

### Influence of soil moisture on urban climate

**Fig. 4:**
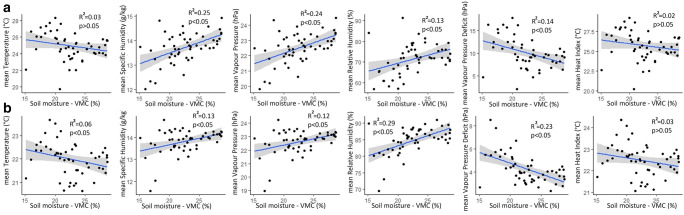
Relationship between surface moisture and **a** daytime climate and **b** nighttime climate in Kampala using linear regression analysis

Specific humidity, vapour pressure and relative humidity increased across Kampala with an increase in surface moisture (Fig. 4). An increase in surface moisture resulted in a decline in temperature and vapour pressure deficit. The sensitivity of urban climate to changes in soil moisture showed diurnal variation as evidenced by the significance of the fitted models and coefficients of determination (Fig. 4). Greater levels of sensitivity to changes in soil moisture were observed for nighttime temperature, daytime specific humidity, daytime vapour pressure, nightime relative humidity and nighttime vapour pressure deficit. However, heat index marginally declined with an increase in surface moisture.

**Fig. 5:**
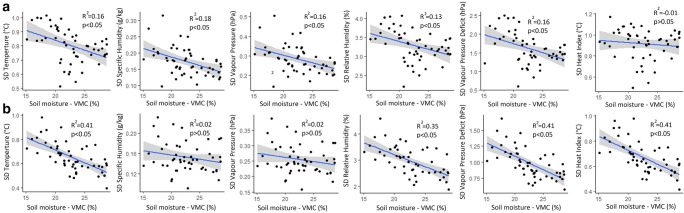
Relationship between spatial differences in **a** daytime climate and **b** nighttime climate in Kampala using linear regression analysis

**Fig. 6:**
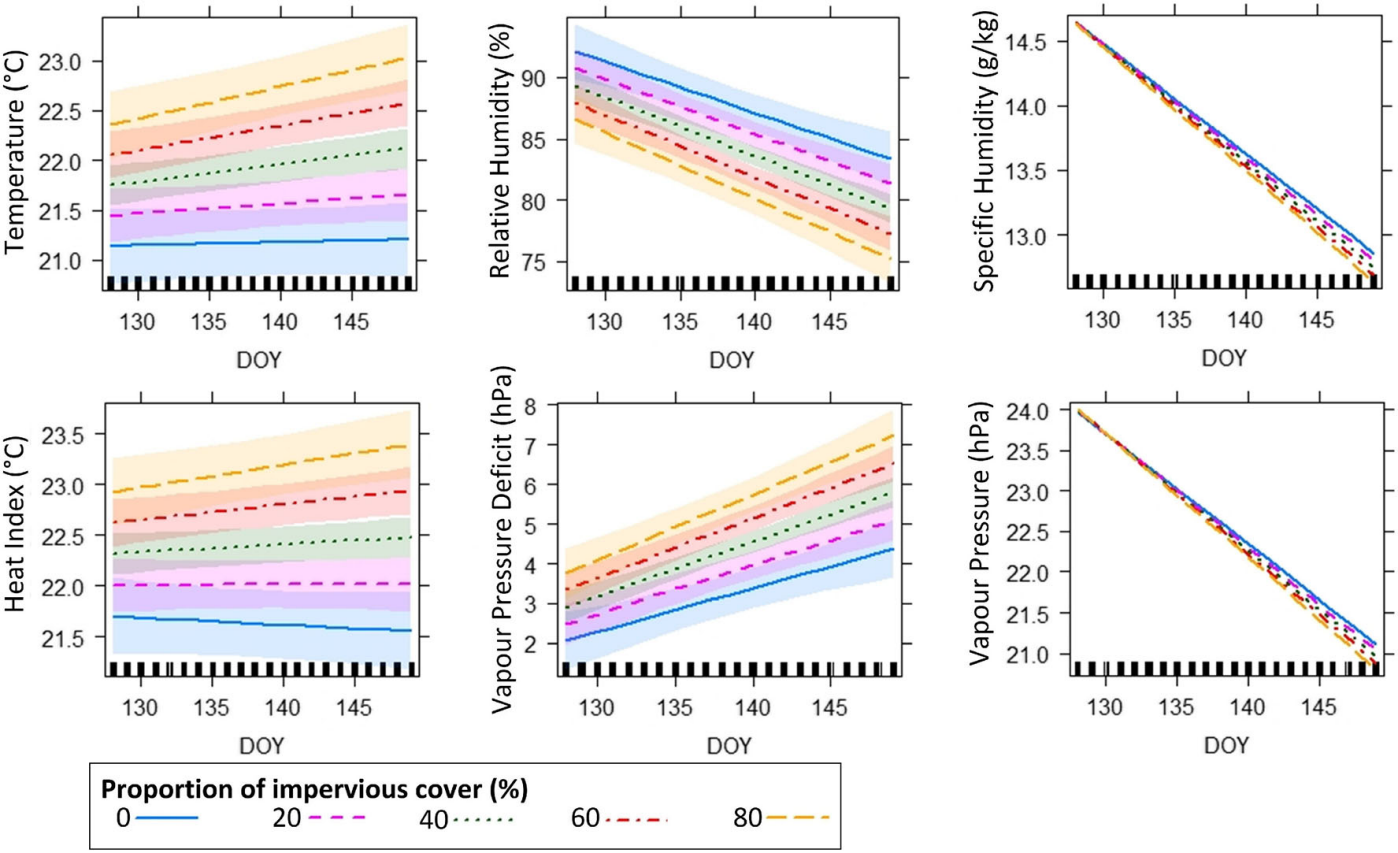
Effect plots showing modelled (linear mixed models) temporal changes in urban climate (with 95% confidence bands) in relation to the proportion of human-made features with the advancement of the dry season. Urban climate variables include nighttime temperature, nighttime heat index, nighttime relative humidity, nighttime vapour pressure deficit, daytime vapour pressure and daytime specific humidity that showed high spatial variation with change in surface moisture

Increase in surface moisture across Kampala resulted in a decline in spatial differences in urban climate (Fig. 5). Moreover, changes in spatial differences in urban climate varied diurnally. The urban climate variables whose spatial differences were most affected by changes in surface moisture include nigttime temperature, daytime specific humidity, daytime vapour pressure, nighttime relative humidity, nighttime vapour pressure deficit and nighttime heat index. The linear mixed models showed that the most built-up locations experienced the fastest changes in nighttime temperature, heat index, relative humidity, vapour pressure deficit, specific humidity and vapour pressure (Fig. 6; Fig A3 in supplementary material). The most built-up locations (approximately 80 % impervious cover) showed the highest temperature, highest vapour pressure deficit, highest heat index, lowest specific humidity, lowest relative humidity and lowest vapour pressure across the dry season.

### Influence of land cover on urban climate

Two candidate models predicting the spatial pattern of nighttime temperature in each season had ΔAIC < 2 (Table 2). The proportion of pervious cover featured in all candidate models for nighttime temperature and had RIV that were more than twice as high as all other variables (Table 3). The combined effect of the proportion of pervious and tree cover significantly influenced nighttime temperature during both seasons. Specifically, nighttime temperature increased with a decline in the proportion of pervious cover and trees (Table 4).

**Table 2:** Results for regression models for determinants of urban climate during the wet and dry season

Variable	Season	Model variables	AICc	logLik	df	*R*^2^	*p*	Delta	Weight
Temperature	Wet	Pervious	21.248	− 6.87	3	64.8	1.13E−05	0	0.692
		Pervious, trees	22.864	− 6.1	4	65.51	4.57E−05	1.616	0.308
	Dry	Pervious	23.89	− 8.19	3	65.05	1.06E−05	0	0.723
		Pervious, trees	25.804	− 7.57	4	65.24	4.88E−05	1.914	0.277
Specific humidity (SH)	Wet	Intercept only	− 20.032	12.37	2			0	1
	Dry	Intercept only	− 20.897	12.8	2			0	0.294
		Paved	− 20.206	13.85	3	5	0.1747	0.691	0.208
		Pervious	− 20.012	13.76	3	4.1	0.1961	0.885	0.189
		Trees	− 19.735	13.62	3	2.7	0.232	1.163	0.165
		Buildings	− 19.469	13.48	3	1.4	0.2742	1.428	0.144
Vapour pressure	Wet	Intercept only	0.183	2.26	2			0	1
	Dry	Intercept only	− 1.458	3.08	2			0	0.44
		Paved	0.097	3.7	3	0.8	0.2976	1.555	0.202
		Pervious	0.261	3.62	3	− 0.03	0.332	1.719	0.186
		Trees	0.435	3.53	3	− 0.9	0.3746	1.894	0.171
Relative humidity (RH)	Wet	Pervious, trees	74.648	− 31.99	4	76.16	1.98E−06	0	0.498
		Paved, buildings	75.675	− 32.5	4	74.91	3.06E−06	1.027	0.298
		Pervious	76.439	− 34.47	3	71.15	1.82E−06	1.791	0.204
	Dry	Pervious, trees	74.515	− 31.92	4	79.4	5.72E−07	0	0.659
		Pervious	75.833	− 34.17	3	75.65	3.85E−07	1.318	0.341
Vapour pressure deficit (VPD)	Wet	Pervious, trees	38.661	− 14	4	74.85	3.12E−06	0	0.563
		Paved, buildings	39.165	− 14.25	4	74.21	3.86E−06	0.504	0.437
	Dry	Pervious, trees	40.324	− 14.83	4	77.65	1.14E−06	0	0.642
		Paved, buildings	41.496	− 15.41	4	76.3	1.88E−06	1.172	0.358
Heat index (HI)	Wet	Pervious	22.233	− 7.37	3	64	1.39E−05	0	0.378
		Paved	23.279	− 7.89	3	62.07	2.26E−05	1.046	0.224
		Paved, buildings	23.475	− 6.4	4	65.38	4.72E−05	1.241	0.203
		Pervious, trees	23.549	− 6.44	4	65.25	4.87E−05	1.316	0.196
	Dry	Pervious	24.502	− 8.5	3	64.3	1.29E−05	0	0.427
		Paved	26.011	− 9.26	3	61.51	2.58E−05	1.509	0.201
		Pervious, trees	26.061	− 7.7	4	65.12	5.02E−05	1.559	0.196
		Paved, buildings	26.279	− 7.81	4	64.74	5.51E−05	1.777	0.176

**Table 3:** Relative importance values (RIV) of determinants of urban climate

Urban climate	Season	Paved surface	Pervious surface	Buildings	Trees
Temperature	Wet	0.28	0.71	0.23	0.23
Temperature	Dry	0.24	0.76	0.21	0.21
SH	Wet	0.169	0.158	0.158	0.168
SH	Dry	0.255	0.232	0.196	0.216
AVP	Wet	0.16	0.162	0.162	0.16
AVP	Dry	0.218	0.201	0.18	0.192
RH	Wet	0.29	0.71	0.36	0.45
RH	Dry	0.18	0.82	0.28	0.47
VPD	Wet	0.37	0.62	0.42	0.45
VPD	Dry	0.28	0.72	0.36	0.48
HI	Wet	0.43	0.57	0.25	0.23
HI	Dry	0.37	0.62	0.23	0.22

**Table 4:** Estimated regression parameters, standard errors (in brackets) and significance levels for the influence of the proportion of pervious and tree cover on urban climate

	Dependent variable							
	Temperature		Relative humidity		Vapour pressure deficit		Heat index	
	(Wet)	(Dry)	(Wet)	(Dry)	(Wet)	(Dry)	(Wet)	(Dry)
Pervious surfaces	− 0.027***	− 0.030***	0.112***	0.129***	− 0.049***	− 0.041***	− 0.029***	− 0.027***
	(0.007)	(0.008)	(0.026)	(0.026)	(0.011)	(0.011)	(0.008)	(0.007)
Trees	− 0.008	− 0.008	0.056**	0.053*	− 0.025**	− 0.025**	− 0.009	− 0.009
	(0.007)	(0.008)	(0.026)	(0.026)	(0.011)	(0.010)	(0.008)	(0.007)
Intercept	22.749***	23.151***	75.497***	72.481***	9.286***	8.132***	23.616***	23.259***
	(0.186)	(0.200)	(0.678)	(0.676)	(0.287)	(0.276)	(0.201)	(0.189)
*R*^2^	0.691	0.689	0.787	0.816	0.800	0.775	0.688	0.689
Adjusted *R*^2^	0.655	0.652	0.762	0.794	0.777	0.749	0.651	0.653
*F* statistic (df = 2; 17)	19.046***	18.829***	31.354***	37.616***	34.010***	29.274***	18.739***	18.840***

*p < 0.1; **p < 0.05; ***p < 0.01

There were no models explaining the spatial patterns of daytime specific humidity and atmospheric vapour pressure in the wet season (Table 2), and all predictor variables had low relative importance values (Table 3). In the dry season, however, the proportion of pervious and tree cover featured in the candidate models for the spatial variation in specific humidity and vapour pressure (Table 2; Fig A4 in supplementary material) and the two predictor variables had higher RIV scores in the dry than the wet season.

The proportion of trees, buildings, pervious cover and paved surfaces featured in the top candidate models predicting the spatial pattern of relative humidity (ΔAIC < 2). However, there were variations in the main predictor variables between the wet and dry seasons (Table 2). All predictor variables featured in the candidate models for vapour pressure deficit in both seasons. Proportions of pervious surfaces had the highest RIV score, followed by the proportion of tree cover for both vapour pressure deficit and relative humidity (Table 3). Increase in proportion of pervious cover and trees was associated with an increase in relative humidity and decline in vapour pressure deficit (Table 4). All predictor variables featured in the candidate models predicting heat index with pervious cover recording the highest RIV score. An increase in the proportion of pervious and tree cover resulted in a decline in heat index (Table 4).

## Discussion

We empirically examined the spatiotemporal dynamics of urban climate in Kampala during the wet to dry season transition using a network of 22 locations that varied in terms of surface cover and structure (0 to 84% human-made impervious surfaces). This study showed the synergistic effect of seasonal changes in surface moisture and land cover composition on microscale climate. The effect of land cover composition on intra-urban climatic differences varied with changes in surface moisture. While gradual increases in surface moisture resulted in a decline in intra-urban climatic differences, diminishing water availability intensified intra-urban climatic differences. The most built-up locations experienced the fastest changes in urban climate. The proportion of pervious surfaces and trees accounted for spatial differences in temperature, heat index, relative humidity and vapour pressure deficit during both seasons. Specific humidity and vapour pressure had an association with land cover only in the dry season. Higher coefficients of determination for the relationship between land cover composition and urban climate were observed for relative humidity and vapour pressure deficit. We also observed diurnal differences for changes in urban climate and its variation across Kampala.

Spatial differences in urban climate in relation to the proportion of human-made features were observed at all points in time. In contrast to less built-up locations, heavily built-up areas experienced higher nighttime temperature, high heat index, high vapour pressure deficit and lower humidity (i.e. vapour pressure and specific humidity) at any given point in time. Similar findings have been observed in regard to spatial variation in temperature (Cavan et al. 2014; Feyisa et al. 2014), humidity (Adebayo 1991; Yang et al. 2017; Hao et al. 2018; Luo and Lau 2019) and heat index (Hass et al. 2016; Scott et al. 2017). Materials such as concrete and asphalt and the presence of buildings promote the transformation of shortwave radiation into heat and its retention (Landsberg 1981; Oke 1987). The absence of vegetation in cities limits latent heat flux from greater evapotranspiration, resulting in warmer temperatures (Chow and Roth 2006; Cavan et al. 2014; Feyisa et al. 2014; Duarte et al. 2015). Additionally, a high proportion of impervious cover restricts water capture and storage, leading to drier neighbourhoods (Whitford et al. 2001). Stronger intra-urban climatic differences with the advancement of the dry season allude to much greater intra-urban climatic differences at the peak of the dry season. This finding is consistent with other studies in tropical urban environments that have observed stronger UHIs (Chow and Roth 2006; Balogun and Balogun 2014; Ojeh et al. 2016; Amorim and Dubreuil 2017; Acero and Gonzalez-Asensio 2018) and UDIs in the dry season (Adebayo 1991). The novelty in this study, however, is that we establish these changes at a high temporal resolution (daily variation) in regard to changes in water availability. Moreover, the current study emphasises the importance of microscale climatic differences due to differences in land cover composition in a tropical urban context.

The high heterogeneity in soil type and depth in Kampala restricted our use of VMC data taken from the topmost soil layer to characterise spatiotemporal patterns of soil moisture in relation to land cover composition. Nonetheless, spatiotemporal patterns of surface moisture with respect to urban form could be inferred from measures of humidity as has been done in the case of the UDI effect (Hao et al. 2018; Luo and Lau 2019). Moreover, humidity has a positive relationship with moisture flux (Archibald and Scholes 2007; Yang et al. 2017; Cai et al. 2019). Therefore, higher humidity in lightly built-up locations indicates higher water capture and storage in comparison to heavily built-up urban areas that experience high water loss through runoff (Whitford et al. 2001). Additionally, the gradual increase in humidity and reduction in temperature with an increase in surface moisture show that increased (adequate) soil moisture enhances latent heat flux through evapotranspiration in Kampala. Soil moisture partitions incoming solar and longwave radiation into outgoing longwave radiation, latent, sensible and ground heat flux and higher moisture content would enhance latent heat flux (Lakshmi et al. 2003; Weng et al. 2004; Berland et al. 2017) in the case of Kampala. Heat and moisture fluxes during the wet season are water-limited because adequate water availability supports evapotranspiration (Pablos et al. 2016; Berland et al. 2017). During the dry season, however, the rapid increase in temperature in the heavily built-up locations occurs as a result of lower latent heat flux due to lower surface water content and high potential evapotranspiration (high sensible heat capture) (Zipper et al. 2017). This contrasts with slow increases in temperature in the lightly built-up locations. High water availability in lightly built-up locations sustains evapotranspiration (latent heat flux) over longer periods. The differences in temperature change between heavily and lightly built-up locations during the dry season highlight contrasts in energy and moisture fluxes due to differences in land cover composition and water availability. Moreover, higher plant water requirements due to increased potential evapotranspiration in heavily built-up locations (Zipper et al. 2017) are exacerbated by low water availability leading to low leaf production and high leaf loss in the most built-up locations (Kabano et al. 2020). Due to spatial differences in canopy cover, the variability in thermal regulation via evapotranspiration further intensifies intra-urban differences in temperature with the advancement of the dry season.

Relative humidity is often overlooked because of its sensitivity to temperature and that the two variables mirror one another. In this study, the combined effect of the proportion of pervious cover and trees accounted for more than 75% of the spatial differences in relative humidity for season averaged data (i.e. both wet and dry seasons). However, we observed much lower coefficients of determination (about 65%) for the effect of pervious cover and trees on temperature, showing the inherent differences between the temperature and relative humidity. Intra-urban differences in temperature are influenced by other variables, including cloud cover, calm conditions and wind (Roth 2012) that were not covered under the scope of this study. Relative humidity is controlled by moisture flux, and surface evaporation, and the relationship between temperature and relative humidity is (temporal) scale-dependent (Yang et al. 2017). Yang et al. (2017) observed that the relationship between monthly and seasonal mean relative humidity and temperature was weak despite hourly relative humidity almost mirroring air temperature. Hao et al. (2018) observed that the decline in relative humidity in the urbanisation process of the Yangtze River Delta had no direct association with changes in air temperature. Moreover, seasonal temperature and relative humidity have been observed to exhibit varying relationships with leaf development (Do et al. 2005; Archibald and Scholes 2007; Jochner et al. 2013) indicating inherent differences between the two variables when examined at a seasonal scale.

## Conclusion

This study provides important evidence about the effect of changes in surface moisture and land cover composition on climate in a city with a tropical rain forest (equatorial) climate type. Although temperature is often the focus of urban climate research, this work addresses the need to include humidity, heat index and water availability to better understand how plant development and heat exposure might be affected by land cover seasonally. Moreover, this work emphasises the importance of examining microscale urban climate processes in relation to neighbourhood characteristics.

In Kampala, the most built-up locations (80% of impervious cover) show the highest heat index and temperature, which are exacerbated with the advancement of the dry season. Our findings show that urban residents in the most built-up parts of the city are the most vulnerable to heat stress, heat-related health complications (e.g. asthma, air pollution and allergens) and reduced productivity at work (Hass et al. 2016). Moreover, high leaf loss and low leaf production of trees due to low water availability and high temperature in the most built-up locations (Kabano et al. 2020) could further elevate the urban heat during the dry season through reduced rates of thermal cooling and reduced provision of shade. Such information about the relationship between neighbourhood characteristics and micro-scale urban climate could be used by urban planners in Kampala and other fast-developing tropical cities in the Global South to design climate-sensitive cities for improved human health and to maximise the benefits of urban vegetation.

## Supplementary Information

ESM 1 (DOCX 0.98 mb)
