# Supplementary material for: Spatiotemporal dynamics of urban climate during the wet-dry season transition in a tropical African city
Source: Int J Biometeorol. 2021 Jan 6;66(2):385–96. doi: 10.1007/s00484-020-02061-1 (PMC8807437; doi:10.1007/s00484-020-02061-1)
Supplement: Supplementary file 1 — (DOCX 0.98 mb) [file 484_2020_2061_MOESM1_ESM.docx]

**International Journal of Biometeorology**

**Electronic Supplementary Material for**

**SPATIOTEMPORAL DYNAMICS OF URBAN CLIMATE DURING THE WET-DRY SEASON TRANSITION IN A TROPICAL AFRICAN CITY**

Peter Kabano^a,b^*, Angela Harris^a^, Sarah Lindley^a^,

^a^*Department of Geography, School of Environment, Education & Development, The University of Manchester, UK*

*^b^Department of Urban and Regional Planning and Geo-information Management, Faculty of Geo-Information Science and Earth Observation (ITC), University of Twente, Enschede, The Netherlands.*

ptkabano@gmail.com; sarah.lindley@manchester.ac.uk; angela.harris@manchester.ac.uk

**Corresponding Author**: Peter Kabano


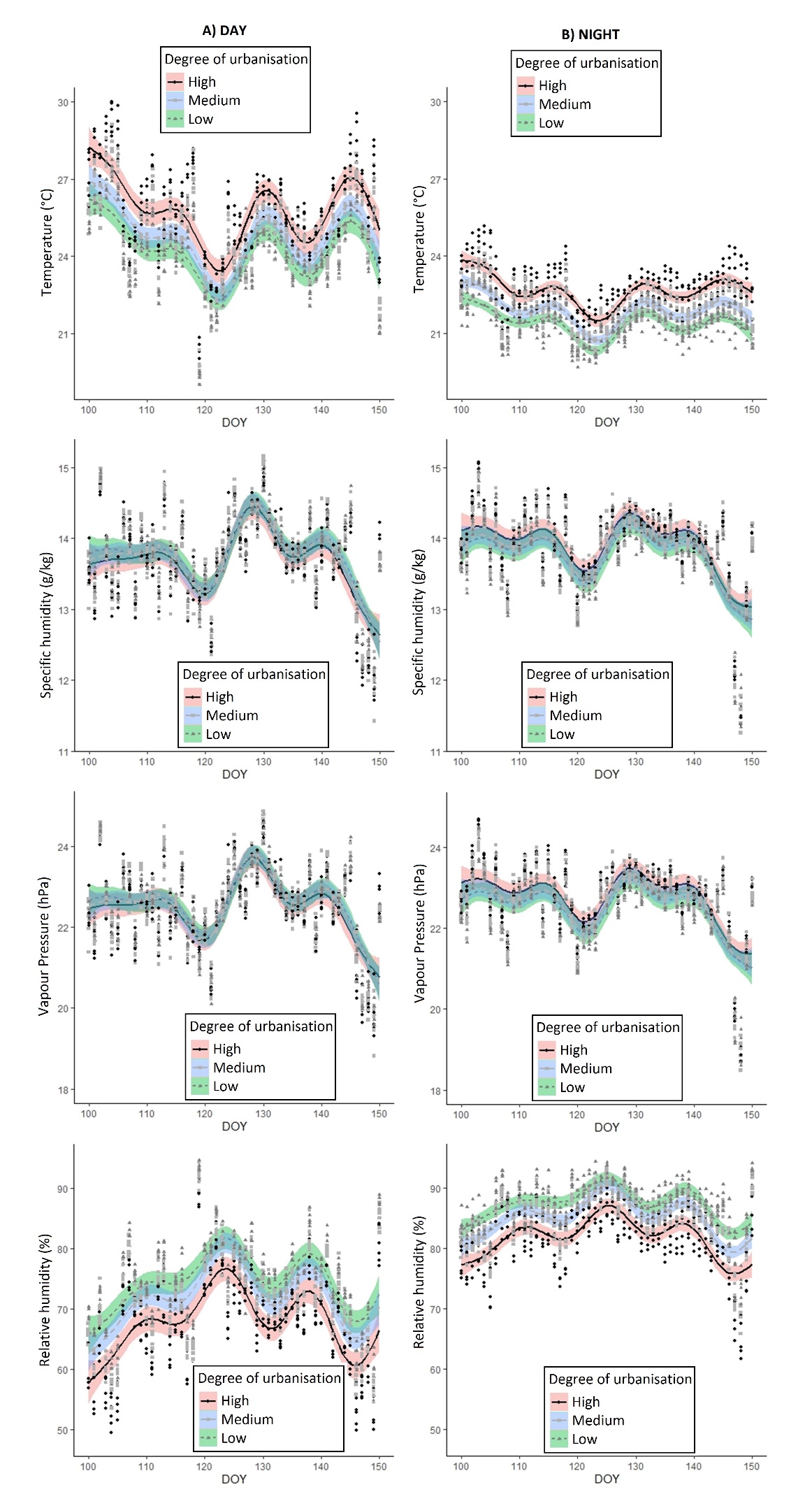


Fig. A1 Time series for day and night time temperature, specific humidity, vapour pressure and relative humidity in relation to the degree of urbanisation intensity across 50 days representing the transition from the wet to dry season


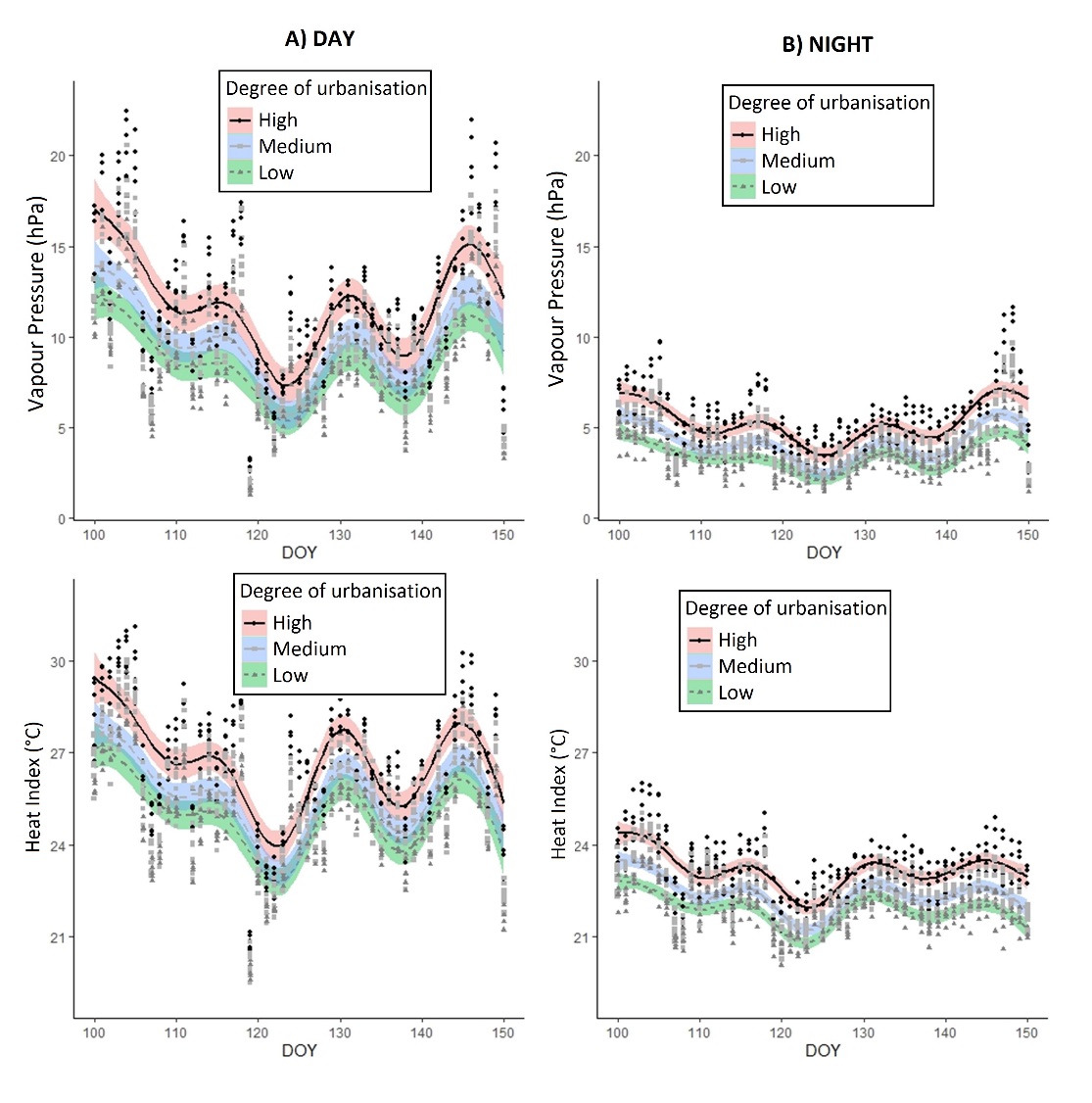


Fig. A2 Time series for day and night time Vapour Pressure Deficit and Heat Index in relation to the degree of urbanisation intensity across 50 days representing the transition from the wet to dry season


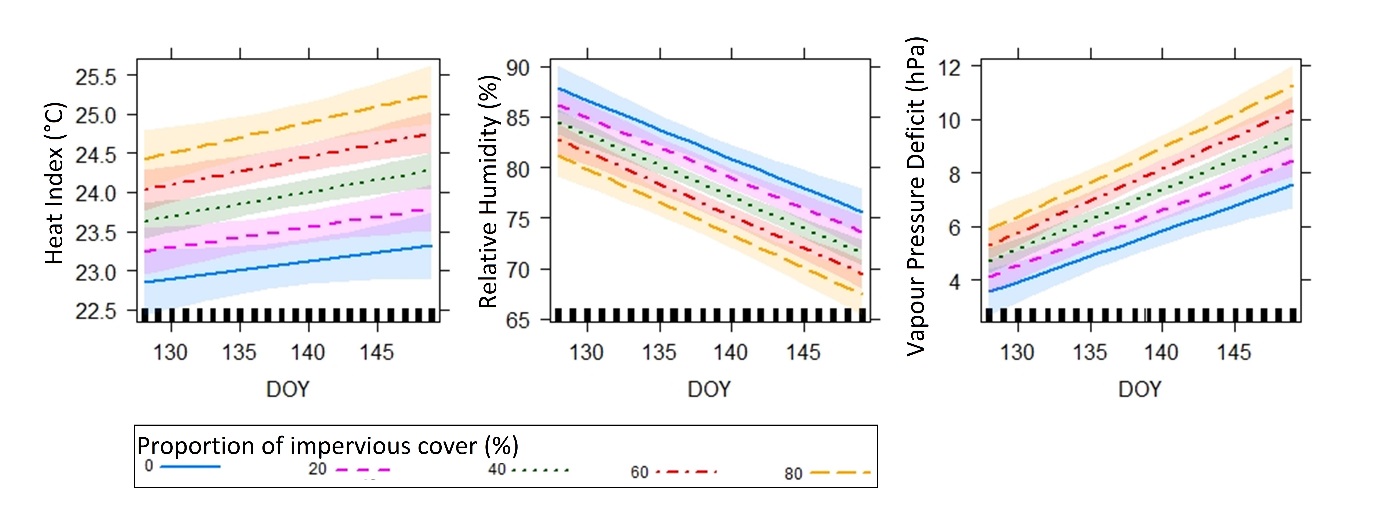


Fig. A3 Effects plots for modelled (using linear mixed models) temporal change of urban climate (daily heat index, relative humidity and vapour pressure deficit) in relation to the proportion of impervious human-made features with the advancement of the dry season


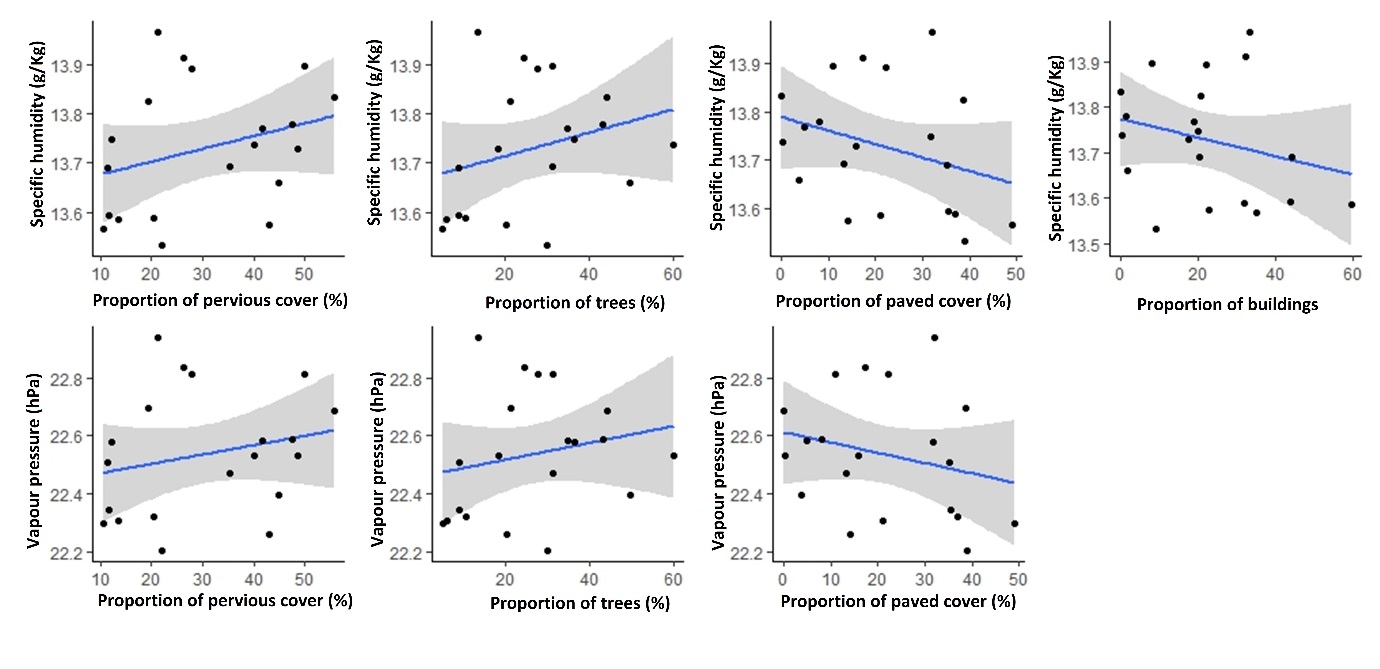


Fig. A4 Linear relationship between land cover and humidity during the dry season. Table 2 in the main manuscript contains relevant statistics
